# Metastatic Clear Cell Carcinoma of Unknown Primary Origin in an Elderly Female Patient With Paraneoplastic Hypercalcemia

**DOI:** 10.7759/cureus.52457

**Published:** 2024-01-17

**Authors:** Bader I Al Omour, Wajeeha Aiman, Gopikrishna Venkatesvaran, Michael Maroules, Gunwant Guron

**Affiliations:** 1 Hematology and Oncology, Saint Michael's Medical Center, Newark, USA; 2 Internal Medicine, Saint Michael's Medical Center, Newark, USA; 3 Internal Medicine, Saint Mary's General Hospital, Passaic, USA; 4 Hematology and Oncology, Saint Mary's General Hospital, Passaic, USA

**Keywords:** ovarian clear cell carcinoma, clear cell cancer, clear renal cell carcinoma, cancer of unknown origin, metastatic cancer of unknown primary

## Abstract

Metastatic clear cell carcinoma (mCCC) is a rare histological subtype of cancer with ovarian and renal origins most common primary sites. Cancer of unknown primary origin (CUP) is a rare type of cancer in the United States and the most common histologic subtypes are adenocarcinoma, squamous cell cancer, and neuroendocrine cancer. We are presenting a rare case of an 86-year-old female patient with mCCC of unknown origin, biopsy and staining showed renal and ovarian in the differential of primary cancer type. However, the patient did not survive the aggressive nature of mCCC and was unable to get any trials of chemotherapy. Primary sites of adenocarcinoma of unknown origin are most common in the breast, lung, pancreas, prostate, colon, and liver. In most cases, empiric chemotherapy with platinum-based agents is the standard of care but needs more data to manage CUP, making it difficult to identify the primary site.

## Introduction

Metastatic clear cell carcinoma (CCC) is a rare and aggressive histological subtype of cancer, with renal and ovarian origins being the most common primary sites. Identifying the primary tumor site in metastatic disease remains crucial for appropriate management and treatment. Of the total, 1-2% of cancers in the United States are cancers of unknown primary (CUP), and the most common histologic subtypes are adenocarcinoma, squamous cell cancer, and neuroendocrine cancer [1]. This report presents a challenging case of an elderly female with a fairly complex medical history presenting with bilateral pulmonary nodules and paraneoplastic hypercalcemia, later diagnosed as metastatic clear cell CUP. In addition, we provide a comprehensive review of the literature on the pathogenesis, prognosis, and management of clear cell carcinoma [2].

## Case presentation

An 86-year-old female with a past medical history of hypothyroidism, bladder prolapse, hypertension, and skin squamous cell carcinoma (SCC) status post-surgical excision five years ago was admitted for evaluation of a syncopal episode and fall, which occurred occasionally for her. She denied any head injury, seizures, weight loss, palpitations, shortness of breath, or chest pain. The patient had no smoking, illicit drug use, or alcohol drinking history. Vital signs were within normal limits: temperature 97.7 F, blood pressure 140/70 mmHg, pulse rate 70 beats per minute (bpm), respiratory rate 18 breaths per minute (bpm), and oxygen saturation 95%. Physical examination was significant for a 5x5 mm scar in the left nasal fold consistent with prior history of squamous cell carcinoma, unremarkable breast and skin exam, and otherwise unremarkable findings.

**Figure 1:**
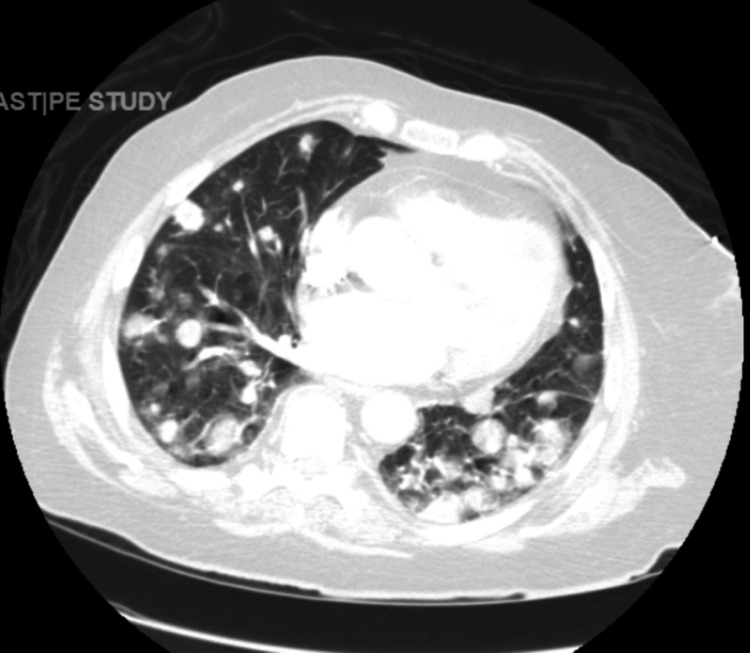
Computed tomographic presentation of pulmonary nodules with bilateral pulmonary edema

Laboratory findings revealed mild anemia (hemoglobin (Hb) 11.2 g/dL), elevated serum calcium (12.3 mg/dL), low parathyroid hormone (PTH), and elevated PTH-related peptide (PTH-rp) suggestive of paraneoplastic hypercalcemia, and mildly elevated aspartate aminotransferase (AST, 45 U/L). PTH, PTH-rp, vitamin D, and imaging studies were done as a diagnostic workup for hypercalcemia. Computed tomography (CT) of the head was unremarkable for acute findings. A chest X-ray demonstrated bilateral pulmonary nodules suggestive of malignancy, later confirmed by a CT scan of the chest (Figure 1).

**Figure 2:**
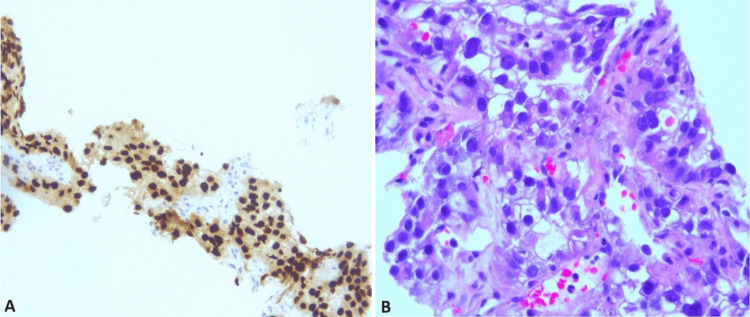
Lung nodule biopsy A depicts CK 7 IHC stain positivity, and B shows clear cancer cells with a high power field. IHC: Immunohistochemical stain, CK: Cytokeratin.

Further investigations with a CT scan of the abdomen and pelvis revealed thickening in the anorectal and gastric regions and cirrhosis. Both colonoscopy and esophagogastroduodenoscopy (EGD) failed to show any malignancy. Subsequently, a lung nodule biopsy was performed, and histopathological analysis revealed metastatic clear cell carcinoma, likely of renal or ovarian origin. The immunohistochemical (IHC) stain profile is exhibited in Figure 2.

As per IHC stains CK7-positive/CK20-negative gives a differential of lung adenocarcinoma, while PAX 8 positivity is highly suspicious for renal, thyroid, ovarian, metastatic RCC, and endometrial adenocarcinoma (Table 1).

**Table 1:** Immunohistochemical stain

Markers	Results
CK 7	Positive
CK 20	Negative
TTF-1	Negative
Napsin 8	Focally Positive
PAX8	Strongly Positive
CD10, GATA3, ER, PR	Negative

A PET scan was done after discharge from the hospital and it showed numerous avid (SUV 8) nodules of varying size in bilateral lungs, multiple mildly enlarged/avid (SUV 9) retroperitoneal and left pelvic sidewall nodes, 2.9 cm oval structure left pelvic sidewall with SUV 9 and distal rectum avid (SUV 10). Bowel can have physiological avidity, but this is quite intense and focal. However, a recent colonoscopy was negative. The patient had uterine prolapse and avidity (SUV 7) in the anterior aspect of the prolapsed segment.

**Figure 3:**
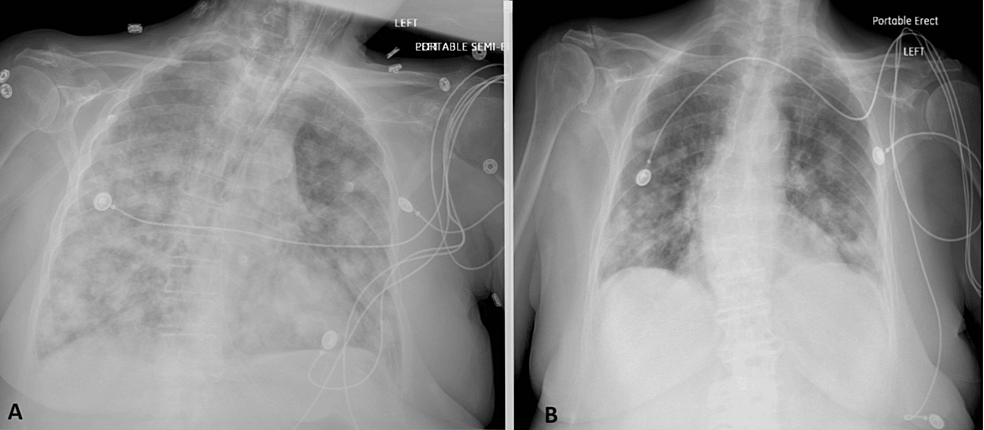
CXR images Image A shows intubated with new pulmonary infiltrates during the second admission on top of lung nodules. B CXR on the first day of previous admission with pulmonary nodules without infiltrates. CXR: Chest X-ray

The patient was admitted again one week after the previous admission to the ICU for the management of severe symptomatic hypercalcemia with metabolic encephalopathy and hypoxic respiratory failure. She was started on normal saline for the recurrence of hypercalcemia from poor oral intake and inactivity. Her fluids were increased, the patient remained obtunded and the corrected Ca for hypoalbuminemia was 15.4. She received pamidronate and calcitonin. The patient was hypoxic requiring BiPAP (a ventilating device) to maintain O_2_ saturation, which shows the worsening of her respiratory function from previous admission (Figure 3).

Hypercalcemia was improving with IV pamidronate, but metastatic disease worsened her respiratory failure and the patient ended up with intubation and mechanical ventilation. Which was complicated by bilateral pneumothorax and chest tube placement, followed by hemodynamic instability and renal failure despite maximal support and the patient expired on the fifth day of the second admission.

## Discussion

The identification of the primary site in metastatic CCC is crucial for tailoring targeted therapy and determining prognosis. In this case, the patient's history of SCC created a bias at the start as SCC metastatic recurrence was top in the differential, complicating the diagnostic process. However, the histopathological analysis of the lung nodule biopsy indicated a distinct tumor entity, leading to further investigations of potential primary sites.

Hypercalcemia, a common clinical presentation in malignancies, prompted initial suspicion of an underlying malignancy in this patient. Hypercalcemia in malignancies is usually attributed to humoral factors, such as PTH-rP secreted by tumor cells or bone metastases. In this case, the presence of hypercalcemia along with the bilateral pulmonary nodules reinforced the likelihood of metastatic malignancy.

Pathogenesis of clear cell carcinoma varies depending on the primary site, but some shared molecular features have been identified. For example, Von Hippel-Lindau (VHL) gene alterations are frequently observed in renal clear cell carcinoma, leading to increased hypoxia-inducible factor (HIF) activity and subsequent angiogenesis, cell proliferation, and tumor growth [3].

Although renal and ovarian origins are the most common primary sites for CCC, the current diagnostic workup, including CT scans and endoscopic evaluations, did not conclusively reveal the primary malignancy. The differential diagnosis of metastatic clear cell cancer CUP should consider other possible primary sites such as the adrenal gland and endometrium, which is aggressive in clear cell carcinoma subtype with intraperitoneal, pelvic, lymphovascular, and lung metastasis, or even the lung, which may be challenging to identify in imaging studies.

The prognosis of metastatic CCC varies depending on the primary tumor site, stage at diagnosis, and response to treatment. Generally, CCC has a poorer prognosis compared to other histological subtypes due to its aggressive behavior and resistance to conventional chemotherapy. Renal clear cell carcinoma, for example, has a 5-year survival rate of approximately 12% for metastatic disease, while ovarian clear cell carcinoma has a 5-year survival rate of 20-25% for advanced stages [4].

The management of metastatic CCC of CUP remains a clinical dilemma due to the lack of evidence-based guidelines. A palliative care discussion was started with the patient's family on 2nd admission. The end-of-life care goal discussion should have started earlier in this case. Due to an unidentified primary source, these discussions were delayed. Treatment strategies for CCC typically depend on the identified primary site and may involve a combination of surgical resection, chemotherapy, and targeted therapies. In ovarian clear cell carcinoma, platinum-based chemotherapy is the mainstay of treatment, but targeted therapies such as bevacizumab (a monoclonal antibody targeting vascular endothelial growth factor) are being investigated in clinical trials [5,6].

## Conclusions

Hypercalcemia presentation of metastatic clear cell carcinoma is a rare presentation of a rare, aggressive type of cancer with unknown primary origin, and management is usually supportive and according to the suspect of the primary site. The presence of advanced age and comorbidities might preclude aggressive intervention, warranting a multidisciplinary approach to determine the most suitable management plan. Treatment options may include palliative care, focusing on symptom management, and maintaining the quality of life with individualized decision-making and close monitoring for potential complications. Some studies have investigated the role of empiric chemotherapy with a platinum-based regimen, as this is the standard treatment for most adenocarcinomas. However, further research is needed to determine the optimal treatment strategy in the absence of an identified primary site. 

## References

[1] Bell CW, Pathak S, Frost P (1989). Unknown primary tumors: establishment of cell lines, identification of chromosomal abnormalities, and implications for a second type of tumor progression. Cancer Res.

[2] Varadhachary GR (2007). Carcinoma of unknown primary origin. Gastrointest Cancer Res.

[3] Huang T, Wang J, Liu R (2023). Safety and efficacy of second-line tki plus anti-pd1 in metastatic non-clear cell renal cell carcinoma: a real-world study. Clin Genitourin Cancer.

[4] Kramer C, Lanjouw L, Ruano D (2024). Causality and functional relevance of BRCA1 and BRCA2 pathogenic variants in non-high-grade serous ovarian carcinomas. J Pathol.

[5] Ito K, Nakagawa M, Shimokawa M (2023). Phase II study of gemcitabine, cisplatin, and bevacizumab for first recurrent and refractory ovarian clear cell carcinoma Kansai Clinical Oncology Group-G1601. Anticancer Drugs.

[6] Otsuka I (2023). Primary retroperitoneal carcinomas: new insights into pathogenesis and clinical management in comparison with ovarian carcinomas and carcinoma of unknown primary. Cancers (Basel).

